# Treatment-Resistant Atopic Dermatitis in Adulthood: A Case Report

**DOI:** 10.7759/cureus.94667

**Published:** 2025-10-15

**Authors:** María Laura Alvarado Fernández, María Luisa Alvarado Mora, Fiorella Apuy Rodríguez, Jessica Arias, Paula Vanegas Navarro

**Affiliations:** 1 Faculty of Medicine, Universidad de Costa Rica, San José, CRI

**Keywords:** adult dermatitis, atopic dermatitis, managing atopic dermatitis, skin disease/dermatology, treatment-resistant dermatitis

## Abstract

Atopic dermatitis (AD) is a chronic and relapsing skin condition that often begins in childhood but can persist or worsen into adulthood, significantly impacting quality of life. We present the case of a 41-year-old woman with long-standing, treatment-resistant AD, unresponsive to various systemic therapies including corticosteroids, methotrexate, thalidomide, and cyclosporine. Despite some control with methotrexate, she experienced frequent flares and side effects. This report highlights the clinical challenges and therapeutic considerations in managing chronic, treatment-resistant AD. It also shows the potential of targeted biologic therapies as more effective and better-tolerated alternatives. Early recognition of treatment resistance is crucial to improving patient outcomes.

## Introduction

Atopic dermatitis (AD) is a skin condition that poses a significant public health concern due to its occurrence in various regions and the resulting rise in healthcare use and expenses [1]. AD is a skin disease characterized by inflammation, intense pruritus, chronicity and relapses that usually occur together with other conditions such as allergic rhinitis, asthma and type I allergies [2]. Symptoms of AD typically begin in childhood, and a small proportion of adults have active disease, although the condition can develop at any stage of life [1]. Approximately 20% of children will develop AD, and only about 3% of adults are typically affected in high-income nations; however, international studies have reported adult prevalence ranging from 2% to 6.9%. More recent analyses found one‑year prevalence rates of 10.2% and 7.2% among adults aged 18 to 85 years; moderate‑to‑severe cases account for 15% to 50% of all occurrences approximately [1,3].

Even though systemic therapies have been studied in recent years, topical therapies for AD remain the primary treatment due to their favorable safety profile and established track record [4]. Although topical treatments allow many patients to manage their symptoms effectively, they are often insufficient for those with moderate to severe AD, who may fail to achieve adequate disease control despite proper use [2].

In the same way, phototherapy is considered when optimized topical treatments fail. If previous options do not provide adequate disease control, systemic immunomodulatory agents are indicated [5]. Systemic immunosuppressants, such as methotrexate or cyclosporine, have historically been used in these cases but are limited by adverse effects and variable efficacy [2]. 

In recent studies on AD, a better understanding of its immunopathogenesis has led to the use of biologic agents for moderate to severe cases. This growing knowledge of the disease supports the development of targeted therapies, which have emerged as promising options for patients with refractory disease [2].

This case report describes the clinical presentation of severe, treatment-resistant AD in an adult patient, highlighting the therapeutic limitations of conventional systemic agents. It also explores the potential role of biologic therapies in achieving sustained disease control.

## Case presentation

A 41-year-old woman from Costa Rica with a past medical history of allergic rhinitis (treated with nasal beclomethasone), asthma (on salbutamol and inhaled beclomethasone), hypertension (on enalapril), and endometriosis (managed with oral contraceptives) presented with long-standing AD. She reported a maternal family history of hypertension, diabetes mellitus, and eczema.

Her dermatologic symptoms began in childhood with eczema affecting flexural regions, neck, periocular areas, upper lip, wrists, thighs, and lower back. She was diagnosed with AD at that time, with notable exacerbation during hot weather.

During early adulthood, her condition worsened significantly, with the development of widespread erythematous, elevated, and intensely pruritic lesions. She initially received systemic corticosteroids for one month, followed by high-dose methotrexate, which achieved disease control for approximately two years. The methotrexate was gradually tapered, but she subsequently experienced new flares. Thalidomide was administered for 6-12 months without significant improvement. Cyclosporine was trialed for less than one year but was discontinued due to worsening hypertension. Despite these interventions, she experienced frequent relapses.

Currently, the patient has been on methotrexate (15 mg weekly) for approximately three years, despite experiencing adverse effects such as nausea and headaches. She also uses antihistamines but continues to have frequent flares. Given the extent and refractoriness of her disease, initiation of biologic therapy is under consideration.

Her condition significantly impacts her quality of life, particularly due to nocturnal pruritus and poor sleep. She has identified heat and certain fabrics as major triggers.

**Figure 1:**
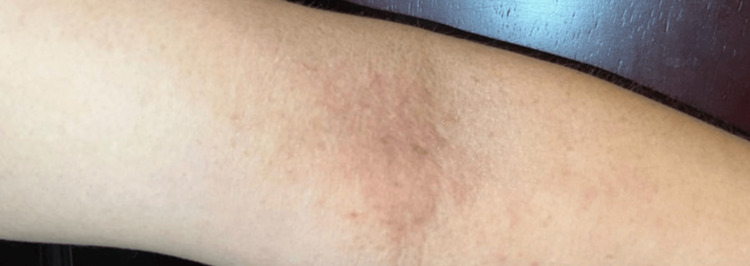
Erythematous-desquamative plaque on the flexural area of the right upper limb, with lichenification and xerosis.

**Figure 2:**
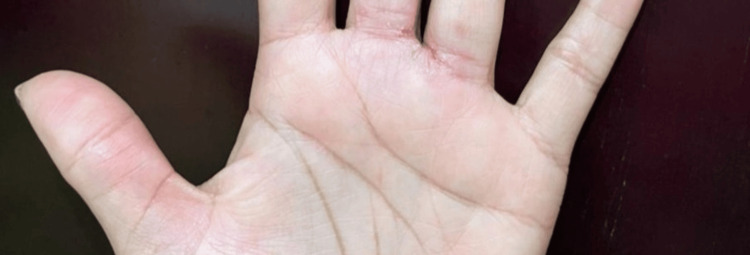
Dyshidrotic dermatitis on the left hand and hyperlinear palm.

Physical examination revealed dyshidrotic eczema on the left hand, erythematous-scaly plaques on upper extremity flexures, xerosis, periorbital darkening, and lichenification of the neck and arms (Figures 1, 2).

## Discussion

The diagnosis of AD is primarily clinical and relies on a combination of typical lesion morphology, distribution, chronicity, and personal history. It has been shown that multiple atopic conditions are linked to AD. Because AD often progresses into other allergic diseases, it is thought that AD represents the beginning of the atopic march. These conditions include asthma, food allergies, and allergic rhinitis. Although the 1980 Hanifin and Rajka criteria are considered the best available diagnosis method, in practice, a simplified clinical approach is often used. The Hanifin and Rajka criteria require 3 minor criteria of 23 and 3 major criteria of 4 for the diagnosis of AD [6].

Since 1980, the Hanifin and Rajka criteria have been the most cited diagnostic tool for AD. The major criteria include dermatitis with classical distribution and morphology, pruritus, a personal or family history of atopy, and a relapsing or chronic course. Minor criteria include xerosis, early age of onset, hyperlinear palms, hand and foot dermatitis, susceptibility to cutaneous infections, pruritus induced by sweating, facial erythema, and aggravation by environmental factors, among others [7]. In our patient, the diagnosis was supported by early childhood onset, chronic relapsing course, flexural involvement, and a family history of atopy, fulfilling the major criteria. She also presented multiple minor criteria, including xerosis, hyperlinear palms, and pruritus worsened by heat.

In a different manner, only three tools commonly used in AD research have been properly validated to assess severity scoring; they are Eczema Area and Severity Index (EASI), Scoring Atopic Dermatitis, and Patient-Oriented Eczema Measure. These instruments are mainly intended for research purposes and are not practical for everyday clinical use [2].

Due to the absence of practical severity scoring tools for clinical use, the Scientific Committee proposed a definition for moderate-to-severe AD based on body surface area (BSA), lesion characteristics and distribution, and quality-of-life impact. They suggest diagnosing moderate-to-severe AD when BSA exceeds 10%, when there is significantly impaired quality of life, when individual lesions show moderate or severe characteristics, or when the affected areas are highly visible or involve functionally important regions such as the face, soles, or palms [2]. Although formal scoring was not performed in our patient, her disease would be classified as moderate-to-severe by clinical judgment due to widespread lesions, functional impact, and significant quality-of-life impairment.

On the other hand, the Scientific Committee outlined types of treatment failure in AD. These include minimal or insufficient clinical improvement, inability to maintain long-term disease control, such as recurring flares, failure to improve quality of life, and adverse events that result in treatment discontinuation. Each of these situations reflects a lack of adequate treatment response, also referred to as treatment resistance [2]. Our patient exemplifies treatment resistance; she experienced multiple flares despite systemic therapy and adverse effects with methotrexate and had to discontinue cyclosporine due to hypertension.

As previously mentioned, the first-line treatment for AD includes environmental modifications, emollients, and conventional topical therapy. When these measures fail to achieve disease control, phototherapy is recommended [5]. In patients with severe or treatment-resistant AD, systemic immunosuppressive therapy is often necessary. Non-biologic systemic agents such as corticosteroids, cyclosporine, azathioprine, mycophenolate mofetil, and methotrexate are commonly used. These medications work by suppressing the immune response, specifically by reducing the expression of cytokines that are proinflammatory and by lowering inflammatory cells [8].

Current literature indicates that azathioprine, cyclosporine, mycophenolate mofetil, and methotrexate are the most commonly used and effective systemic treatments for AD. In contrast, other options such as leukotriene inhibitors and oral calcineurin inhibitors have limited supporting evidence [5].

Cyclosporine is a well-known and effective option for treating AD that does not respond to topical therapy [5]. However, it is not recommended for use longer than one year due to significant side effects such as an increased risk of lymphoma, infections, tremors, hypertension, nephrotoxicity, and others [2]. This limitation became clinically relevant in our patient, as hypertension led to early discontinuation of cyclosporine.

Likewise, methotrexate is also used for refractory AD [5]. It reaches its maximum effect at 10 weeks; beyond 12 to 16 weeks, no further improvement is observed even with higher doses. This medication has serious adverse effects such as pulmonary fibrosis, increased risk of skin cancer, bone marrow suppression, and others [2]. Our patient developed gastrointestinal discomfort and headaches on methotrexate but remained on low-dose therapy due to a lack of alternative options.

In recent years, biologic therapies have advanced rapidly, offering targeted treatment by focusing on specific inflammatory mediators. Clinical trials and case reports have demonstrated that blocking key cytokines involved in the pathogenesis of AD is an effective therapeutic strategy. These agents offer the advantage of fewer side effects, less frequent laboratory monitoring, and simpler dosing regimens [8]. There is a consensus supporting the use of biologic therapies as systemic treatment for patients with severe AD who are refractory to or intolerant of topical treatments. Currently, dupilumab and tralokinumab are approved in the United States for this indication [9].

## Conclusions

We report this case because AD is less frequent in adults compared to children, and moderate to severe forms constitute an important clinical burden. This case of a patient from Costa Rica illustrates the complex and often challenging journey faced by patients and physicians in managing chronic, severe, and treatment‑resistant AD. It highlights the need for personalized therapeutic strategies, as conventional systemic treatments may not provide durable disease control for every patient. Close clinical monitoring, patient‑centered education, and emotional support are essential to improve quality of life. Importantly, this case supports the early consideration of biologic therapies in refractory adult AD, offering the potential for better disease control and outcomes. Sharing real‑world experiences like this one contributes to expanding clinical understanding and guiding future care for similar patients.
